# Comparative evaluation of liraglutide plus metformin combination therapy versus metformin monotherapy in patients with type 2 diabetes mellitus: A retrospective clinical study

**DOI:** 10.1097/MD.0000000000047562

**Published:** 2026-02-13

**Authors:** Tingting Mai

**Affiliations:** a Department of Endocrinology, Heyuan People’s Hospital, Heyuan, Guangdong Province, China.

**Keywords:** glucagon-like peptide-1 receptor agonist, glycated hemoglobin, insulin resistance, liraglutide, metformin, type 2 diabetes mellitus

## Abstract

Durable glycemic control remains challenging in patients with type 2 diabetes mellitus inadequately controlled with metformin alone. Liraglutide, a glucagon-like peptide-1 receptor agonist, has been proposed as a beneficial add-on therapeutic option. This retrospective study included adults with type 2 diabetes mellitus treated between March 2021 and March 2025. Patients were divided into a combination group receiving liraglutide plus metformin and a control group receiving metformin alone. Glycemic parameters and safety outcomes were assessed at baseline and after 6 months of treatment. Statistical comparisons were conducted using Welch *t* test and Chi-square or Fisher exact test. A total of 207 patients were analyzed. Compared with metformin monotherapy, combination therapy achieved greater reductions in glycated hemoglobin (‐1.8 ± 0.9% vs ‐1.3 ± 0.8%; *P* < .001), fasting and postprandial plasma glucose, fasting insulin, and homeostasis model assessment of insulin resistance. The proportion achieving composite glycemic targets was higher in the combination group (76.5% vs 57.9%; *P* = .006). Adverse event incidence and treatment discontinuation did not differ significantly between groups. Liraglutide add-on therapy provided superior glycemic improvement compared with metformin alone and demonstrated acceptable safety and tolerability.

## 1. Introduction

Type 2 diabetes mellitus (T2DM) remains a major global public health burden, with steadily increasing prevalence, accelerated disease progression, and substantial long-term complications despite advances in screening, pharmacologic therapeutics, and individualized risk management strategies. International epidemiological analyses indicate that the continuous rise in T2DM incidence is strongly associated with obesogenic nutritional patterns, sedentary lifestyle, population aging, as well as metabolic and inflammatory dysregulation, resulting in significant healthcare expenditure and premature disability-adjustedlife years loss worldwide.^[1,2]^ Glycemic control remains the core therapeutic objective in T2DM management because chronic hyperglycemia contributes to microvascular and macrovascular complications, including diabetic nephropathy, neuropathy, retinopathy, cardiomyopathy, and cerebrovascular events, and achieving persistent glycemic normalization confers substantial long-term clinical benefit. Metformin is recognized as first-line pharmacotherapy for T2DM based on its robust efficacy, safety, weight neutrality, and affordability. However, a substantial proportion of patients fail to achieve sustained glycemic control with metformin monotherapy, especially in real-world clinical care where metabolic heterogeneity, progressive β-cell dysfunction, and declining insulin secretory capacity are common. For this population, rational second-line combination strategies are required. In recent guidelines and consensus statements, glucagon-like peptide-1 receptor agonists (GLP-1RAs), such as liraglutide, are increasingly recommended as preferred combination therapy due to their glucose-dependent insulinotropic effect, suppression of glucagon overproduction, beneficial effects on body weight, low intrinsic hypoglycemia risk, and favorable cardiometabolic profile.^[3–5]^

Recent high-quality trials and real-world evidence have demonstrated that GLP-1RA add-on therapy enhances glycated hemoglobin (HbA1c) reduction and improves postprandial glucose excursions more effectively than traditional drug intensification approaches while minimizing the need for additional glucose-lowering agents and excessive polypharmacy.^[6]^ Additionally, GLP-1RA therapy exerted beneficial effects on insulin resistance, metabolic efficiency, satiety signaling, gastrointestinal hormone regulation, and systemic inflammatory stress response, all of which are increasingly recognized as key contributors to metabolic disease progression and cardiometabolic risk trajectories. The weight-modulation and cardiovascular protection observed in GLP-1RA class studies further support their integration into contemporary T2DM treatment paradigms, particularly in subpopulations with high cardiometabolic vulnerability.^[7,8]^ Despite evidence supporting the superiority of GLP-1RAs over traditional escalation pathways such as sulfonylureas or insulin in selected clinical scenarios, comparative data directly evaluating liraglutide plus metformin versus metformin monotherapy in routine, non-trial practice conditions remain relatively limited, particularly in real-world East Asian cohorts, where insulin resistance profiles, β-cell reserve, and dose–response characteristics may differ from Western populations. Furthermore, treatment intensification patterns and safety signals under real practice conditions require additional evaluation to guide rational regimen selection and refine personalized pharmacologic management strategies. Therefore, further clinical research examining real-world therapeutic effectiveness, metabolic benefit, and tolerability is necessary to provide stronger evidence for optimized stepwise pharmacotherapy in T2DM.

The present study focuses on evaluating glycemic improvement, insulin resistance reduction, treatment intensification needs, and short-term safety outcomes in patients with T2DM receiving liraglutide plus metformin combination therapy compared with metformin monotherapy. Findings generated from clinically relevant populations may provide important implications for clinical decision-making, real-world treatment selection, and evidence-based refinement of individualized T2DM therapeutic pathways.

## 2. Methods

### 2.1. Study design

This study was approved by the Ethics Committee of Heyuan People’s Hospital. The present study was designed as a retrospective including patients with type 2 diabetes mellitus who received treatment at our institution between March 2021 and March 2025. Eligible participants were adults with a confirmed diagnosis of type 2 diabetes mellitus based on standard clinical and laboratory criteria and had complete baseline and follow-up clinical data. Exclusion criteria included patients with type 1 diabetes mellitus, gestational diabetes, acute metabolic decompensation at presentation, severe hepatic or renal dysfunction, active malignancy, severe cardiovascular or cerebrovascular events within the preceding 3 months, or those who discontinued therapy or had missing outcome data. According to the therapeutic regimen received, patients were stratified into the observation group, in which individuals received combination therapy with liraglutide and metformin, and the control group, in which patients were treated with metformin alone. All study procedures were performed in compliance with the ethical principles outlined in the Declaration of Helsinki, and protocol approval was obtained from the Institutional Medical Ethics Committee. Written informed consent was obtained from all participants.

### 2.2. Treatment protocols

Patients in the observation group received combination therapy consisting of subcutaneous liraglutide in addition to oral metformin. Liraglutide was initiated at a dose of 0.6 mg once daily and titrated to 1.2 to 1.8 mg once daily based on glycemic response and individual tolerability. Metformin was administered at a total daily dose of 1500 to 2000 mg, divided into 2 or 3 doses, with dose adjustments performed according to gastrointestinal tolerance and renal function indicators. Antidiabetic therapy was maintained continuously throughout the treatment course, and follow-up evaluations were conducted at predetermined clinical visits.

Patients in the control group received metformin monotherapy. The initial metformin dose was 500 mg 2 to 3 times daily, and treatment could be escalated to a total daily dose of 1500 to 2000 mg according to efficacy and tolerability, following the same strategy of dosing adjustment and safety monitoring as in the observation group. No glucagon-like peptide-1 receptor agonists or other glucose-lowering agents with similar mechanisms of action were used during the treatment period in this group.

For both groups, lifestyle counseling based on standardized diabetes management recommendations, including dietary guidance, exercise recommendations, and self-monitoring of blood glucose, was provided as routine clinical care and was not considered part of the study intervention. Continuous clinical monitoring, adverse event screening, and laboratory assessments were performed in accordance with institutional clinical practice standards.

### 2.3. Data collection and follow-up

Clinical data were obtained retrospectively through extraction from the hospital electronic medical record system. Baseline demographic characteristics, clinical history, medication records, anthropometric parameters, laboratory indicators, and comorbidities were collected at treatment initiation. Laboratory parameters included HbA1c, fasting plasma glucose (FPG), postprandial plasma glucose (PPG), fasting insulin concentration, lipid metabolic indicators, and renal function parameters. The homeostasis model assessment of insulin resistance (HOMA-IR) was calculated using the formula: HOMA-IR = fasting insulin (μU/mL) × fasting glucose (mmol/L)/22.5. Information on antidiabetic drug dosage, dose modification, initiation of additional glucose-lowering medication, and treatment-related adverse events was also recorded. All laboratory tests were performed in the institutional clinical laboratory using standardized biochemical testing methods and validated automated instruments. All enrolled patients were followed continuously for a total treatment duration of 6 months. Follow-up assessments were conducted at predetermined time points through outpatient clinical visits, electronic medical record review, and additional telephone contact when needed. Glycemic indices, metabolic parameters, and medication usage were reassessed during follow-up, and treatment safety and tolerability outcomes were continuously monitored. Final endpoint data were collected at the completion of the 6-month follow-up to ensure consistent and comparable outcome evaluation across both treatment groups.

Given the retrospective and non-randomized design of this study, several measures were implemented to minimize potential bias and confounding. First, baseline demographic and clinical characteristics were systematically compared between groups to assess initial comparability. Second, uniform inclusion and exclusion criteria were applied, and all patients were followed over an identical 6-month period to reduce time-related bias. Third, laboratory measurements were performed using standardized methods in a single institutional laboratory.

Nevertheless, residual confounding related to unmeasured factors such as lifestyle adherence, socioeconomic status, and physician prescribing preference cannot be fully excluded. These limitations may affect causal inference, and the results should therefore be interpreted as associative rather than definitive evidence of treatment superiority.

### 2.4. Statistical analysis

All statistical analyses were performed using Statistical Package for the Social Sciences software version 28.0 (IBM Corp., Armonk) and R statistical software version 4.3.2 (R Foundation for Statistical Computing, Vienna, Austria). Continuous variables were examined for normality using the Shapiro–Wilk test. Normally distributed data were expressed as mean ± standard deviation. Comparisons between the 2 treatment groups were conducted using Welch *t* test to account for potential variance heterogeneity. Categorical variables were presented as frequencies and percentages, and intergroup comparisons were performed using the Chi-square test or Fisher exact test when expected frequencies were less than 5. Treatment effect was evaluated by calculating within-group changes (end of follow-up minus baseline), and between-group differences in mean changes were compared using Welch *t* test. Ninety-five percent confidence intervals were calculated for effect estimates. Missing data were handled using complete case analysis. Statistical significance was defined as a two-sided *P* value <.05.

## 3. Results

### 3.1. Baseline characteristics

In total, 207 patients were enrolled, including 81 patients in the observation group and 126 patients in the control group. Baseline demographic and clinical characteristics were comparable between the 2 groups. The mean age was 56.1 ± 8.4 years in the observation group and 56.8 ± 8.3 years in the control group (*P* = .557). Body mass index showed similar distribution between groups (28.3 ± 3.6 kg/m^2^ vs 28.1 ± 3.5 kg/m^2^, *P* = .693). Parameters reflecting diabetes severity were also balanced, including HbA1c (8.7 ± 1.2% vs 8.8 ± 1.3%, *P* = .571), fasting plasma glucose (9.9 ± 2.0 mmol/L vs 10.1 ± 2.1 mmol/L, *P* = .491), and HOMA-IR (7.0 ± 3.2 vs 7.2 ± 3.4, *P* = .669). Blood pressure, lipid profiles, and renal function indicators such as estimated glomerular filtration rate (85.9 ± 18.9 mL/min/1.73 m^2^ vs 84.7 ± 19.6 mL/min/1.73 m^2^, *P* = .660) were also not statistically different between the 2 cohorts. Likewise, the distribution of sex, hypertension, dyslipidemia, and current smoking status did not differ significantly (all *P* > .05). These results demonstrate that baseline metabolic conditions and cardiovascular risk factors were balanced across groups prior to treatment initiation (Table 1).

**Table 1:** Baseline characteristics of the study population.

Variable	Observation (n = 81)	Control (n = 126)	Test statistic	*P*-value
Age (yr)	56.1 ± 8.4	56.8 ± 8.3	*t* = −0.59	.557
Body mass index (kg/m^2^)	28.3 ± 3.6	28.1 ± 3.5	*t* = 0.39	.693
Duration of T2DM (yr)	8.2 ± 4.4	8.0 ± 4.5	*t* = 0.32	.752
HbA1c (%)	8.7 ± 1.2	8.8 ± 1.3	*t* = −0.57	.571
Fasting plasma glucose (mmol/L)	9.9 ± 2.0	10.1 ± 2.1	*t* = −0.69	.491
Postprandial plasma glucose (mmol/L)	12.8 ± 2.8	13.0 ± 2.9	*t* = −0.50	.621
Fasting insulin (μU/mL)	16.2 ± 6.1	15.8 ± 6.3	*t* = 0.46	.649
HOMA-IR	7.0 ± 3.2	7.2 ± 3.4	*t* = −0.43	.669
Systolic blood pressure (mm Hg)	134.6 ± 14.8	135.1 ± 15.2	*t* = −0.24	.814
Diastolic blood pressure (mm Hg)	80.9 ± 8.2	81.1 ± 8.5	*t* = −0.17	.866
Triglycerides (mmol/L)	2.15 ± 0.92	2.20 ± 0.95	*t* = −0.38	.706
Total cholesterol (mmol/L)	5.02 ± 0.96	5.06 ± 0.98	*t* = −0.29	.772
HDL-C (mmol/L)	1.08 ± 0.23	1.06 ± 0.22	*t* = 0.62	.535
LDL-C (mmol/L)	3.05 ± 0.82	3.10 ± 0.85	*t* = −0.42	.673
eGFR (mL/min/1.73 m^2^)	85.9 ± 18.9	84.7 ± 19.6	*t* = 0.44	.660
Male sex, n (%)	45 (55.6)	68 (54.0)	χ^2^ = 0.05	.823
Hypertension, n (%)	39 (48.1)	63 (50.0)	χ^2^ = 0.07	.795
Dyslipidemia, n (%)	44 (54.3)	71 (56.3)	χ^2^ = 0.08	.774
Current smoking, n (%)	21 (25.9)	28 (22.2)	χ^2^ = 0.37	.541

eGFR = estimated glomerular filtration rate, HbA1c = glycated hemoglobin, HDL-C = high-density lipoprotein cholesterol, HOMA-IR = homeostasis model assessment of insulin resistance, LDL-C = low-density lipoprotein cholesterol, T2DM = type 2 diabetes mellitus.

### 3.2. Comparative analysis of HbA1c reduction between liraglutide plus metformin combination therapy and metformin monotherapy

A significant improvement in HbA1c levels was observed in both groups, but the magnitude of reduction was greater in patients receiving liraglutide combined with metformin. HbA1c decreased from 8.7 ± 1.2% to 6.9 ± 0.8% in the observation group and from 8.8 ± 1.3% to 7.5 ± 0.9% in the control group. The mean HbA1c reduction was ‐1.8 ± 0.9% and ‐1.3 ± 0.8% in the observation and control groups, respectively. The between-group difference in the mean change was ‐0.50% (95% confidence interval [CI], ‐0.74 to ‐0.26), and this difference was statistically significant (*t* = ‐4.07, *P* < .001) (Table 2).

**Table 2:** Change in glycated hemoglobin (HbA1c) from baseline to end of follow-up.

Outcome	Observation (n = 81)	Control (n = 126)	Between-group comparison
HbA1c at baseline, % (mean ± SD)	8.7 ± 1.2	8.8 ± 1.3	–
HbA1c at end of follow-up, % (mean ± SD)	6.9 ± 0.8	7.5 ± 0.9	–
Within-group change, % (mean ± SD)	‐1.8 ± 0.9	‐1.3 ± 0.8	–
Mean difference in change (Obs ‐ Ctrl), % (95% CI)	–	–	‐0.50 (‐0.74 to ‐0.26)
Welch *t* test for change	–	–	*t* = −4.07, *P* < .001

CI = confidence interval, HbA1c = glycated hemoglobin, SD = standard deviation.

### 3.3. Comparative assessment of glucose metabolic parameters and achievement of composite glycemic control targets

From baseline to study end, both groups exhibited improvements across glycemic indices, with consistently larger mean reductions in the observation group. FPG decreased by ‐3.0 ± 1.6 mmol/L in the observation group versus ‐2.5 ± 1.5 mmol/L in the control group (mean difference in change, ‐0.50 mmol/L; 95% CI, ‐0.94 to ‐0.06; Welch *t* = ‐2.25; *P* = .026). PPG declined by ‐4.1 ± 2.2 mmol/L versus ‐3.4 ± 2.1 mmol/L, respectively (mean difference, ‐0.70 mmol/L; 95% CI, ‐1.30 to ‐0.10; Welch *t* = ‐2.27; *P* = .024). Fasting insulin fell by ‐3.7 ± 3.6 μU/mL in the observation group and ‐2.0 ± 3.4 μU/mL in the control group (mean difference, ‐1.70 μU/mL; 95% CI, ‐2.68 to ‐0.72; Welch *t* = −3.39; *P* < .001). HOMA-IR decreased by ‐2.7 ± 2.0 versus ‐1.8 ± 2.1 (mean difference, ‐0.90; 95% CI, ‐1.47 to ‐0.33; Welch *t* = −3.10; *P* = .002). The composite glycemic target was achieved by 76.5% in the observation group and 57.9% in the control group, yielding an absolute risk difference of 18.6% (χ^2^ = 7.53; *P* = .006) (Table 3).

**Table 3:** Comparative changes in glycemic and insulin resistance parameters.

Outcome	Group	Baseline (mean ± SD)	End (mean ± SD)	Change (end − base)	MD in change (Obs − Ctrl)	95% CI	Test	*P*-value
FPG (mmol/L)	Observation (n = 81)	9.9 ± 2.0	6.9 ± 1.1	‐3.0 ± 1.6				
	Control (n = 126)	10.1 ± 2.1	7.6 ± 1.2	‐2.5 ± 1.5	‐0.50	‐0.94 to ‐0.06	Welch *t* = −2.25	.026
PPG (mmol/L)	Observation	12.8 ± 2.8	8.7 ± 1.6	‐4.1 ± 2.2				
	Control	13.0 ± 2.9	9.6 ± 1.8	‐3.4 ± 2.1	‐0.70	‐1.30 to ‐0.10	Welch *t* = −2.27	.024
Fasting insulin (μU/mL)	Observation	16.2 ± 6.1	12.5 ± 5.3	−3.7 ± 3.6				
	Control	15.8 ± 6.3	13.8 ± 5.8	‐2.0 ± 3.4	‐1.70	‐2.68 to ‐0.72	Welch *t* = −3.39	<.001
HOMA-IR	Observation	7.0 ± 3.2	4.3 ± 2.2	−2.7 ± 2.0				
	Control	7.2 ± 3.4	5.4 ± 2.5	−1.8 ± 2.1	−0.90	−1.47 to −0.33	Welch *t* = −3.10	.002
Composite glycemic target	Observation	–	–	62/81 (76.5%)				
	Control	–	–	73/126 (57.9%)	+18.6%	–	χ^2^ = 7.53	.006

CI = confidence interval, FPG = fasting plasma glucose, HbA1c = glycated hemoglobin, HOMA-IR = homeostasis model assessment of insulin resistance, PPG = postprandial plasma glucose, SD = standard deviation.

### 3.4. Comparative evaluation of medication adjustment, adverse events, and treatment discontinuation

The safety and tolerability assessment showed that the proportion of patients requiring the initiation of an additional glucose-lowering medication was significantly lower in the observation group compared with the control group (7.4% vs 19.8%; χ^2^ = 5.99, *P* = .014). The incidence of treatment-related adverse events was comparable between groups, with 22.2% in the observation group and 17.5% in the control group, and no statistically significant difference was detected (χ^2^ = 0.72, *P* = .397). Discontinuation due to intolerance occurred infrequently in both cohorts, with 4.9% in the observation group and 1.6% in the control group, and the difference was not statistically significant (Fisher exact *P* = .212) (Table 4).

**Table 4:** Safety and tolerability outcomes during follow-up.

Outcome	Group	Value	Effect/test	*P*-value
Initiation of additional glucose-lowering medication, n/N (%)	Observation	6/81 (7.4)	χ^2^ = 5.99	.014
	Control	25/126 (19.8)		
Any treatment-related AE, n/N (%)	Observation	18/81 (22.2)	χ^2^ = 0.72	.397
	Control	22/126 (17.5)		
Discontinuation due to intolerance, n/N (%)	Observation	4/81 (4.9)	Fisher exact	.212
	Control	2/126 (1.6)		

## 4. Discussion

The present retrospective analysis demonstrated that, over a 6-month treatment period, liraglutide added to metformin achieved greater improvements in glycemic control than metformin alone while maintaining a comparable safety profile. The combination regimen was associated with a larger reduction in HbA1c (between-group mean difference in change, −0.50%; 95% CI, ‐0.74 to ‐0.26; *P* < .001) alongside superior improvements in secondary glycemic indices, including fasting plasma glucose, postprandial plasma glucose, fasting insulin, and HOMA-IR. In parallel, a higher proportion of patients in the combination group reached the prespecified composite glycemic target. Requirements for treatment intensification were lower in the combination group, whereas the incidence of treatment-related adverse events and discontinuation due to intolerance did not differ significantly between groups. Collectively, these findings support the incremental glycemic benefits of GLP-1RA add-on therapy to metformin under routine clinical care conditions and indicate that such benefits can be achieved without an excess safety burden over 6 months. Contemporary guideline statements that prioritize GLP-1RAs as effective options after metformin (particularly when weight reduction and low hypoglycemia risk are desired) are consistent with these observations.

Mechanistically, the observed superiority of the combination regimen in reducing HbA1c and postprandial glycemia is congruent with the known pharmacology of GLP-1RAs: enhancement of glucose-dependent insulin secretion, suppression of inappropriate glucagon secretion, delayed gastric emptying, and consequent mitigation of postprandial excursions. Current standards synthesize these effects into treatment algorithms that recommend GLP-1RA therapy when additional glucose lowering beyond metformin is needed, especially in individuals for whom weight management and avoidance of hypoglycemia are priorities.^[9,10]^ Our data showing greater reductions in fasting insulin and HOMA-IR further suggest an improvement in insulin sensitivity and/or reduced insulin demand, aligning with recent evidence that GLP-1RA therapy favorably modulates metabolic efficiency. The magnitude of HbA1c reduction observed with liraglutide add-on in this cohort falls within the range reported for the GLP-1RA class in recent clinical literature. Although variability arises from baseline HbA1c, dose titration, adherence, and background therapies, our between-group difference in HbA1c change (−0.50%) is directionally consistent with the incremental benefit expected when adding a GLP-1RA to metformin versus continuing metformin alone. These class-consistenteffects are reflected in recent guidance and comparative analyses that position GLP-1RAs among the preferred add-on options after metformin.^[11]^

The improvements in fasting insulin and HOMA-IR provide internal coherence to the primary glycemic findings. While HOMA-IR is an indirect index and subject to physiological and assay variability, the greater reduction observed with liraglutide add-on may indicate decreased insulin requirements owing to enhanced incretin-mediated insulin efficiency and reduced glucagon drive. Recent syntheses have also reported anti-inflammatoryand metabolic benefits with GLP-1RA therapy, which could contribute to improved whole-body insulin sensitivity over intermediate time horizons, though such pathways were not directly measured in this study.^[12,13]^ The safety profile observed here aligns with recent real-world and clinical-trial experience. The rates of “any treatment-related adverse event” did not differ significantly between groups over 6 months, and discontinuations due to intolerance were infrequent. Contemporary real-world effectiveness studies evaluating once-daily liraglutide add-on therapy similarly report acceptable tolerability with gastrointestinal events as the most common adverse effects and low discontinuation rates, supporting the generalizability of our findings to routine practice.^[14]^

Our findings accord with and extend recent evidence regarding the glycemic efficacy and tolerability of GLP-1RAs added to metformin. The 2024 and 2025 updates of the American Diabetes Association Standards of Care summarize high-qualitydata showing that GLP-1RAs produce clinically meaningful HbA1c reductions, favorably affect postprandial glucose, and promote weight loss with a low risk of hypoglycemia; they are recommended as preferred add-on therapy to metformin in many clinical scenarios, including individuals prioritizing weight reduction or those at high cardiovascular risk. The present analysis reproduces the incremental HbA1c benefit of GLP-1RA add-on over 6 months and demonstrates concordant improvements across FPG, PPG, and HOMA-IR. Beyond guidelines, recent comparative and real-world reports provide complementary context.^[15,16]^ A 2023 network meta-analysis comparing multiple GLP-1RAs confirmed class-wide reductions in HbA1c and body weight without an increased incidence of hypoglycemia relative to comparators, supporting their positioning as effective glucose-lowering therapies with ancillary metabolic benefits. Real-world cohorts evaluating liraglutide add-on regimens have further documented clinically relevant HbA1c reductions and acceptable tolerability profiles across diverse practice settings. Our data are consistent with these observations, although the present study was not designed to quantify weight change or gastrointestinal adverse-event subtypes.^[17]^ In addition, emerging analyses underscore that the glycemic and metabolic gains with GLP-1RAs translate into treatment de-intensification in some patients, reflected by reduced need for therapy escalation. In our cohort, the initiation of additional glucose-lowering medication was significantly less frequent with liraglutide plus metformin than with metformin alone, mirroring recent clinical practice insights that GLP-1RA add-on can reduce downstream intensification, at least over intermediate follow-up. Taken together, the current results are directionally aligned with contemporary comparative effectiveness research and guideline-driven care pathways, reinforcing the role of GLP-1RA add-on to metformin as a rational strategy for patients requiring additional glycemic control.

Beyond statistical significance, the present study demonstrates a clinically meaningful incremental reduction in HbA1c of 0.50% with liraglutide add-on therapy compared with metformin alone. Importantly, this benefit was consistently supported by parallel improvements in fasting and postprandial glucose, fasting insulin levels, and HOMA-IR, suggesting a coherent metabolic effect rather than an isolated glycemic change. The reduced need for additional glucose-lowering medications observed in the combination group further underscores the potential of liraglutide to delay treatment intensification in routine practice, which may translate into simplified therapeutic regimens and improved long-term adherence.

For clinicians managing adults with type 2 diabetes inadequately controlled on metformin, adding liraglutide offers incremental HbA1c reduction and broader glycemic improvements without excess adverse events over 6 months. The higher achievement of composite glycemic targets and the lower need for treatment intensification observed in this study suggest potential to simplify regimens and delay escalation, consistent with American Diabetes Association recommendations that favor GLP-1RAs when additional efficacy with weight neutrality/benefit and low hypoglycemia risk is desired. Patient selection should consider gastrointestinal tolerability, injection preference, and access, but the net clinical profile supports routine consideration of GLP-1RA add-on after metformin. This study has several strengths. First, it reflects real-world practice over a uniform 6-month treatment horizon with standardized laboratory testing, allowing consistent ascertainment of primary and secondary glycemic outcomes. Second, the dataset captured multiple domains of glucose metabolism (HbA1c, FPG, PPG, fasting insulin, and HOMA-IR) and clinically relevant endpoints (composite glycemic targets, treatment intensification, and adverse events), enabling convergent assessment of therapeutic effectiveness and tolerability. Third, both treatment groups were comparable at baseline across demographic, metabolic, and comorbidity profiles, reducing confounding by indication at study entry.

Several limitations merit consideration. The retrospective, nonrandomized design is susceptible to residual confounding and selection bias despite baseline balance; unmeasured factors (e.g., diet quality, physical activity, medication adherence, socioeconomic determinants) may have influenced outcomes. The follow-up was limited to 6 months; durability of glycemic benefit and long-term safety, including cardiovascular and renal outcomes, were not evaluated. Weight change, gastrointestinal adverse-event subtypes, and hypoglycemia incidence were not systematically adjudicated, limiting comparisons with trials that include these endpoints. Finally, single-center data may constrain generalizability across health systems with different prescribing patterns and formularies. Prospective randomized studies or pragmatic trials with longer follow-up and broader outcome capture would strengthen causal inference and external validity.

## 5. Conclusions

In this retrospective study, liraglutide combined with metformin produced greater improvements in HbA1c, glucose metabolism parameters, and glycemic target achievement compared with metformin monotherapy, without increasing adverse event incidence. These findings suggest that liraglutide add-on therapy may offer enhanced glycemic benefit with acceptable tolerability in patients with type 2 diabetes inadequately controlled with metformin alone.

## Author contributions

**Conceptualization:** Tingting Mai.

**Data curation:** Tingting Mai.

**Formal analysis:** Tingting Mai.

**Funding acquisition:** Tingting Mai.

**Investigation:** Tingting Mai.

**Writing – original draft:** Tingting Mai.

**Writing – review & editing:** Tingting Mai.

## References

[1] Long Y Zhang Y. Liraglutide combined with metformin treatment for obese people with type 2 diabetes mellitus: a systematic review and meta-analysis. Ir J Med Sci. 2023;192:2809–14. 37036569 10.1007/s11845-023-03337-2

[2] Jacobsen LV Flint A Olsen AK Ingwersen SH. Liraglutide in type 2 diabetes mellitus: clinical pharmacokinetics and pharmacodynamics. Clin Pharmacokinet. 2016;55:657–72. 26597252 10.1007/s40262-015-0343-6 PMC4875959

[3] Capehorn MS Catarig AM Furberg JK. Efficacy and safety of once-weekly semaglutide 1.0mg vs once-daily liraglutide 1.2mg as add-on to 1-3 oral antidiabetic drugs in subjects with type 2 diabetes (SUSTAIN 10). Diabetes Metab. 2020;46:100–9. 31539622 10.1016/j.diabet.2019.101117

[4] Shaman AM Bain SC Bakris GL. Effect of the glucagon-like peptide-1 receptor agonists semaglutide and liraglutide on kidney outcomes in patients with type 2 diabetes: pooled analysis of SUSTAIN 6 and LEADER. Circulation. 2022;145:575–85. 34903039 10.1161/CIRCULATIONAHA.121.055459 PMC8860212

[5] Bendotti G Montefusco L Lunati ME. The anti-inflammatory and immunological properties of GLP-1 receptor agonists. Pharmacol Res. 2022;182:106320. 35738455 10.1016/j.phrs.2022.106320

[6] Kayaniyil S Lozano-Ortega G Bennett HA. A network meta-analysis comparing exenatide once weekly with other GLP-1 receptor agonists for the treatment of type 2 diabetes mellitus. Diabetes Ther. 2016;7:27–43. 26886440 10.1007/s13300-016-0155-1 PMC4801811

[7] Ard J Fitch A Fruh S Herman L. Weight loss and maintenance related to the mechanism of action of glucagon-like peptide 1 receptor agonists. Adv Ther. 2021;38:2821–39. 33977495 10.1007/s12325-021-01710-0 PMC8189979

[8] Yousef CC Thomas A Matar MA. Liraglutide effects on glycemic control and weight in patients with type 2 diabetes mellitus: a real-world, observational study and brief narrative review. Diabetes Res Clin Pract. 2021;177:108871. 34052248 10.1016/j.diabres.2021.108871

[9] Lee J Kim R Kim MH. Weight loss and side-effects of liraglutide and lixisenatide in obesity and type 2 diabetes mellitus. Prim Care Diabetes. 2023;17:460–5. 37541792 10.1016/j.pcd.2023.07.006

[10] Hu EH Tsai ML Lin Y Chou TS Chen TH. A review and meta-analysis of the safety and efficacy of using glucagon-like peptide-1 receptor agonists. Medicina (Kaunas). 2024;60:357. 38541083 10.3390/medicina60030357 PMC10972401

[11] Tegegne BA Adugna A Yenet A. A critical review on diabetes mellitus type 1 and type 2 management approaches: from lifestyle modification to current and novel targets and therapeutic agents. Front Endocrinol (Lausanne). 2024;15:1440456. 39493778 10.3389/fendo.2024.1440456 PMC11527681

[12] Ren Y Chen Y Zheng W. The effect of GLP-1 receptor agonists on circulating inflammatory markers in type 2 diabetes patients: a systematic review and meta-analysis. Diabetes Obes Metab. 2025;27:3607–26. 40230207 10.1111/dom.16366

[13] Karagiannis T Avgerinos I Liakos A. Management of type 2 diabetes with the dual GIP/GLP-1 receptor agonist tirzepatide: a systematic review and meta-analysis. Diabetologia. 2022;65:1251–61. 35579691 10.1007/s00125-022-05715-4 PMC9112245

[14] Sodhi M Rezaeianzadeh R Kezouh A Etminan M. Risk of gastrointestinal adverse events associated with glucagon-like peptide-1 receptor agonists for weight loss. JAMA. 2023;330:1795–7. 37796527 10.1001/jama.2023.19574 PMC10557026

[15] Jensen AB Renström F Aczél S. Efficacy of the glucagon-like peptide-1 receptor agonists liraglutide and semaglutide for the treatment of weight regain after bariatric surgery: a retrospective observational study. Obes Surg. 2023;33:1017–25. 36765019 10.1007/s11695-023-06484-8 PMC9918402

[16] Ma J Fu J Guo N Liu Z. Clinical efficacy and safety of liraglutide and dapagliflozin on glucose and lipid metabolism and insulin function in patients with type 2 diabetes mellitus. Altern Ther Health Med. 2024;30:144–51. 38294744

[17] Xie Z Hu J Gu H Li M Chen J. Comparison of the efficacy and safety of 10 glucagon-like peptide-1 receptor agonists as add-on to metformin in patients with type 2 diabetes: a systematic review. Front Endocrinol (Lausanne). 2023;14:1244432. 37701904 10.3389/fendo.2023.1244432 PMC10493284

